# Evaluating the Diagnostic Value of a Combined Indicator of Vitamin B_12_ Status (cB_12_) Throughout Pregnancy

**DOI:** 10.3389/fnut.2021.789357

**Published:** 2022-01-26

**Authors:** Marie-Joe Dib, Maria Gumban-Marasigan, Rozzie Yoxall, Toby Andrew, Dominic J. Harrington, Agata Sobczyńska-Malefora, Kourosh R. Ahmadi

**Affiliations:** ^1^Department of Nutritional Sciences, School of Biosciences and Medicine, University of Surrey, Guildford, United Kingdom; ^2^Department of Biostatistics and Epidemiology, School of Public Health, Imperial College, London, United Kingdom; ^3^The Nutristasis Unit, Viapath, St. Thomas' Hospital, London, United Kingdom; ^4^Department of Genomics of Common Disease, Imperial College, London, United Kingdom; ^5^Division of Women's Health, School of Medicine, King's College London, London, United Kingdom; ^6^Faculty of Life Sciences & Medicine, King's College London, London, United Kingdom

**Keywords:** cobalamin (Cbl), pregnancy, biomarker, genetic epidemiology, nutrient status, cB_12_, deficiency

## Abstract

**Background:**

Inadequate provision of vitamin B_12_ during pregnancy is associated with a number of adverse maternal and fetal outcomes. We set out to (1) suggest pregnancy-specific reference ranges for a range of biomarkers of vitamin B_12_; (2) assess the temporal behaviors of these markers over the course of pregnancy; and (3) test whether any biomarkers, including the genetic marker *HIBCH rs291466* strongly associated with MMA measured early in pregnancy could reliably and significantly predict future B_12_ status within a healthy UK population of pregnant women.

**Materials and Methods:**

We used existing biobank samples from the placebo arm of the UK Selenium in PRegnancy Intervention (SPRINT) study, to generate biochemical data for serum folate, B_12_, holotranscobalamin (HoloTC), total homocysteine (tHcy), and MMA, calculate cB_12_, and genotyped the polymorphism *rs291466* in gene *HIBCH* on a total of *n*=114 women across trimesters 1–3 of their pregnancy. We performed a series of exploratory cross-sectional and longitudinal analyses to investigate levels at each trimester, suggest references ranges, evaluate changes and correlations between the B_12_ biomarkers, and assess the predictive capabilities of each biomarker from 12-weeks to 35-weeks of gestation.

**Results:**

Significant changes in all vitamin B_12_ biomarker values were observed over the three trimesters (*P* < 0.05). Our study shows that cB_12_ values were largely constant and stable throughout trimester 1 (T1) and T2 (i.e., up to week 20), but declined significantly in T3 (−66% | P < 0.001). Yet, cB_12_ generally remained within the normal boundaries. We identified pregnancy and trimester-specific reference ranges for each biomarker at each trimester, notably for total serum B_12_. This marker fell below the recommended cut-offs in 1/3 of the cohort at the third trimester, contrasting other markers (mostly normal). Our multivariate analyses indicated that none of the biomarkers could reliably and accurately predict any other biomarkers than themselves later in pregnancy. Yet, HoloTC seems to be a promising predictor within the limitations of our cohort, constituted of B_12_-replete individuals. Most notably, cB_12_ did not significantly predict itself between trimesters. Finally, we show that the *HIBCH* variant has little predictive power for MMA or cB_12_ as it does not explain the significant increase in MMA concentrations nor the decline of cB_12_ throughout pregnancy.

**Conclusion:**

Trimester-specific reference ranges for biomarkers of vitamin B_12_ in normal pregnancy are suggested. However, these biomarkers have limited predictive value in identifying mothers at elevated risk of vitamin B_12_ insufficiency/deficiency during pregnancy.

## Introduction

Vitamin B_12_ or cobalamin (Cbl), is an essential human micronutrient which plays a fundamental role as a cofactor in two main cellular reactions: the remethylation of homocysteine (tHcy) to methionine by the enzyme methionine synthase (MS) in the cytoplasm, to facilitate methyl donation via S-adenosylmethionine (SAM) to RNA/DNA, phospholipids and neurotransmitters (1); and the mitochondrial conversion of methylmalonyl-CoA to succinyl-CoA by the enzyme methylmalonyl-CoA mutase (MUT) for energy production.

In the United Kingdom, 12.4% of women of childbearing age (19 to 39 years old) have serum B_12_ (sB_12_) concentrations in the deficient range (<148 pmol/L), and a further 44% in the marginal or insufficient range (150–258 pmol/L) (2). During pregnancy and lactation, maternal requirements for B_12_ become significantly higher and despite efficient enterohepatic recirculation, significant maternal vitamin B_12_ stores (~3,000 μg in women eating an omnivorous diet) and requirements for fetal development of just 50 μg (3, 4), it has been shown that low levels of sB_12_ among pregnant women is very common with a global prevalence of 20–38% (5, 6). In the UK, studies have estimated the prevalence at ~20–34% among white, non-diabetic individuals (7, 8). The risk of B_12_ deficiency may be particularly concerning amongst those consuming minimal or no animal products (9, 10). It is important to note that low sB_12_ values are not necessarily indicative of true B_12_ status, as the major portion of B_12_ in blood circulates bound to haptocorrin (HC), an “inert” protein with a slow turnover rate. Its total concentration decreases during pregnancy, causing an observed decrease in sB_12_ that is not necessarily reflective of a metabolic insufficiency (11). Biochemical analysis of HoloTC rather than total B_12_ is supported in the literature (11).”

Early detection of vitamin B_12_ deficiency or insufficiency during pregnancy is critical because of its association with a myriad of rare and common adverse outcomes that affect both the mother and offspring pre- and postnatally, including recurrent miscarriage, preterm birth (PB) and low birth weight (LBW), poor neurocognitive development, and post-natal depression (3–5, 7, 12–22). At a global scale, PB and LBW alone cause ~34% of the 2.5 million neonatal deaths each year (23). Nevertheless, antenatal screening of vitamin B_12_ is currently piecemeal (24). In the UK there is currently no gold standard test for measuring vitamin B_12_ deficiency (25). Vitamin B_12_ status is still largely assessed through the measurement of plasma/serum B_12_ although other biomarkers, including holotranscobalamin (HoloTC), total homocysteine (tHcy) and methylmalonic acid (MMA), are now available, which have improved sensitivity and specificity of “functional” B_12_ status assessment but are prone to interference from sample matrix effects. Furthermore, depending on a single marker introduces additional risk, as different assays have different vulnerabilities (26). Pregnancy poses particular challenges relating to changes in concentrations of B_12_ biomarkers. These are indicative of altered physiology rather than true biochemical deficiency (11, 27, 28). For example, sB12, the most commonly used indicator of maternal B12 status, has been shown to decrease over the course of a normal pregnancy, even at adequate dietary intakes (3, 27–29). While B_12_ absorption remains unchanged during pregnancy (3, 11, 27, 30, 31), proposed explanations for progressive sB_12_ decline include haemo-dilution, B_12_ redistribution and transportation to the fetus (29, 32).

HoloTC concentrations, in general, tend to remain constant across the three trimesters, and are similar to those of non-pregnant women (11). Other functional biomarkers of B_12_ status, namely MMA and tHcy, have particular behaviors in pregnancy. Whereas, MMA levels have been shown to generally increase during pregnancy (11, 27, 29, 33), tHcy in pregnancy is considerably lower than in non-pregnant women and starts to increase gradually only at around 32–34 GW, reaching the pre-pregnancy values at the time of delivery (32). The Hcy changes in pregnancy are considered to be a result of haemodilution, increased GFR, albumin decline and hormonal changes (hCG, estradiol) and not so much driven by the standard nutritional determinants of Hcy (e.g., folate & B_12_). Women supplemented with folic acid in late pregnancy have lower Hcy than those not supplemented but folic acid does not prevent the increase of Hcy in T3 (34). The data generally indicates that fluctuations in B_12_ status probably has very little influence on tHcy changes during pregnancy (35).

Thus, interpretation of these changes ultimately needs a thorough understanding of the adaptive physiological responses to avoid overdiagnosis of B_12_ deficiency during pregnancy, but also highlights an urgent need for pregnancy-specific reference ranges (36). More recently, a composite score of B_12_ status (cB_12_) has been suggested to be a more useful metric to assess B_12_ status, as it combines biochemical measurements of sB_12_ and HoloTC with functional biomarkers of B_12_ status, MMA and tHcy, while taking into account folate status and age (37, 38). We have previously addressed the strengths and limitations of multiple analyte testing of B_12_ status, and have shown the potential utility of the composite score cB_12_ in population screening of B_12_ status/deficiency in a UK healthy population of adults and older adults (38). However, there have been no studies of B_12_ in pregnancy using the combined indicator score cB_12_.

In this study, we aimed to further develop our understanding of how static—sB_12_, folate, HoloTC—and functional—tHcy, MMA—biomarkers of vitamin B_12_ status as well as cB_12_ behave over the course of healthy pregnancy. Furthermore, we wanted to evaluate if there is any reliable predictive value of such biomarkers to help identify women at the initial stages of pregnancy who may be at elevated risk of vitamin B_12_ insufficiency/deficiency. Finally, we set out to test if the gain of function effects of *HIBCH rs291466* on MMA and cB_12_ variability in the context of pregnancy are the same as those identified by us and others in population settings (38). Understanding the interaction of *rs291466* with MMA and cB_12_ variability during pregnancy could provide us with novel insights into the application of the cB_12_ score as a diagnostic tool of B_12_ deficiency in an ‘at-risk' population, given the current limitations of pregnancy-specific cut-offs for B_12_ biomarkers.

## Materials and Methods

### Study Participants

**Figure 1:**
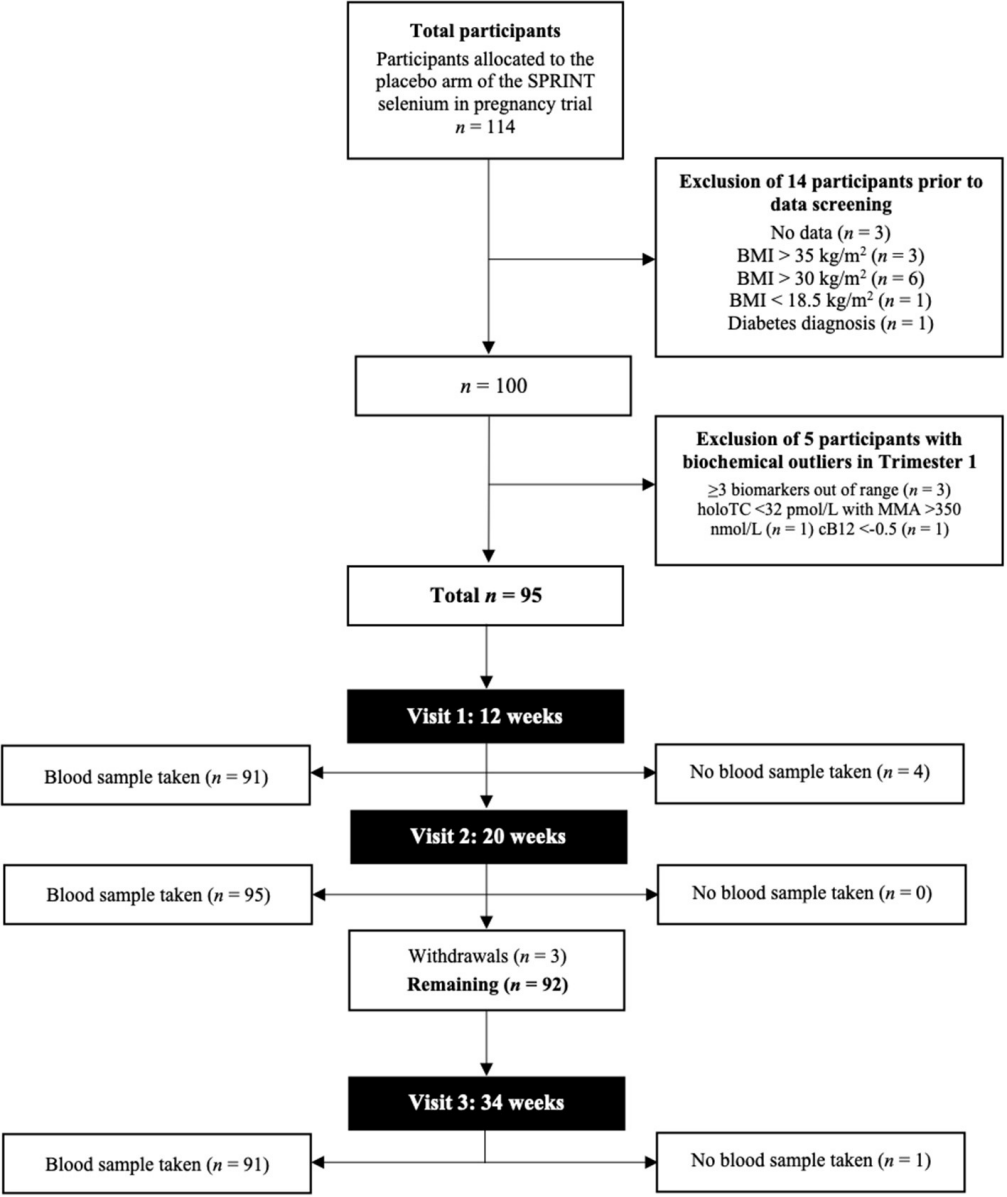
Flowchart of the study cohort selection from the SPRINT study placebo arm. BMI, body mass index; cB_12_, combined indicator of vitamin B_12_ status; HoloTC, holotranscobalamin; MMA, methylmalonic acid.

We used existing biobank samples from the placebo arm of the SPRINT (Selenium in PRegnancy INTervention) trial (39) (www.isrctn.com; ISRCTN37927591). The full selection criteria and recruitment methods of subjects for the study has been previously described (40). Briefly, a total of 114 women were recruited to the original study at their 12-week scan dated between 14th July 2009 and 6th June 2011, while under antenatal care at John Radcliffe Hospital, Oxford, UK. Further to randomization in the original SPRINT study, we excluded 14 out of 114 participants with unavailable samples for analysis for all three trimesters, were diagnosed with diabetes (41), had a body mass index (BMI) in the ‘underweight' (<18.5 kg/m^2^), “obese” (30–34.9 kg/m^2^) and “severely obese” categories (>35 kg/m^2^) (42) (Table 1). We further excluded five participants due to the lack of biochemical data, or if they had measures outside the cut-offs presented in Supplementary Tables 1, 2. A total of 95 women were consequently included in this study (Figure 1). For the SPRINT trial, participants in the placebo arm were required to take yeast-based placebo tablets for the duration of the study, which would have had no impact on B_12_ metabolism or status. The SPRINT study was undertaken according to the Declaration of Helsinki and ethical approval came from the Milton Keynes Research Ethics Committee (reference 08/H0603/46). Written consent was provided as part of the original study.

**Table 1:** Characteristics of the study population^a^.

**Variable**	**Trimester (week of gestation)**			***p*-value (T1–T2)**	***p*-value (T2–T3)**
	**1 (week 12)**	**2 (week 20)**	**3 (week 34)**		
Age, years					
Mean ± SD	31.1 ± 3.52				
Range	22–39				
BMI, kg/m^2^					
Mean ± SD	23.7 ± 2.61	24.9 ± 2.61	27.8 ± 2.89	<0.001	<0.001
Range	18.5–29.7	19.2–31.4	21.8–36.6		
Vitamin B_12_, pmol/L					
Median (IQR)	224 (103)	205 (99)	173 (85)	<0.001	<0.001
Range	108–496	85–475	75–546		
< 148 pmol/L, *n* (%)	8 (10)	11 (12)	32 (35)		
HoloTC, pmol/L					
Median (IQR)	68 (38)	63 (35)	53 (38)	0.001	0.001
Range	28–128	20–128	25–128		
< 32 pmol/L, *n* (%)	2 (2)	3 (3)	7 (8)		
tHcy, μmol/L					
Median (IQR)	4.6 (1.3)	4.4 (1.1)	5.4 (1.9)	0.02	<0.001
Range	3.7–10.1	2.5–9.3	1.0–10.9		
> 15 μmol/L, *n* (%)	–	–	–		
MMA, nmol/L					
Median (IQR)	143 (68)	150 (75)	174 (100)	<0.001	<0.001
Range	45–406	50–582	36–452		
> 350 nmol/L, *n* (%)	2 (2)	2 (2)	5 (6)		
Folate, nmol/L					
Median (IQR)	33.7 (6.2)	22.9 (11.2)	18.1 (17.4)	<0.001	0.005
Range	17.4–47.1	12.0–45.3	5.9–45.3		
<6.8 nmol/L, *n* (%)	–	–	1 (1)		
cB_12_					
Mean ± SD	0.50 ± 0.43	0.50 ± 0.44	0.17 ± 0.43	n.s.	<0.001
Range	−0.29 to 1.62	−0.41 to 1.59	−0.68 to 1.17		
< -0.5, *n* (%)	–	–	5 (5)		
HoloTC: serum B_12_ ratio, %					
Median (IQR)	29 (13)	30 (16)	36 (18)	n.s.	<0.001
Range	14–61	9–62	10–73		

*Reported p-values are from repeated measures ANOVA with post-hoc for cB_12_ and BMI, and Friedman's test with Wilcoxon signed ranks tests for all other variables, for differences between trimesters 1 and 2, and trimesters 2 and 3*.

a *Data are means ± SD, medians (IQR). BMI, body mass index; HoloTC, holotranscobalamin; MMA, methylmalonic acid; tHcy, total homocysteine*.

### Biochemical Data

Laboratory analyses of all biochemical data have been previously outlined in the SPRINT cohort study (40). We generated biochemical data on serum folate, sB_12_, HoloTC, tHcy and MMA. sB_12_, HoloTC and folate were measured using the Architect 2000 Series Analyzer (Abbott Diagnostics). Serum MMA was measured by liquid chromatography tandem mass spectrometry (LC-MS/MS). The laboratory participates in the appropriate EQA schemes and is ISO 15189:2012 accredited for the analysis. In line with standard procedures, the laboratory returned a small number of results (*N* = 5) as above/below linear ranges of the assays. These values were removed from the statistical analyses based on using the continuous variables. cB_12_ was calculated through Fedosov's equation, expressed as: cB_12_ = log_10_ [HoloTC x B_12_/(MMA x tHcy)] – [3.79/1 (age/230)^2.6^] (37). Furthermore, we generated data on the ratio of HoloTC to sB_12_ concentrations [HoloTC: sB_12_]. The ratio is not used diagnostically in clinical settings, except when haptocorrin deficiency is suspected. HC can bind both B_12_ and B_12_ analogs (43); however, sB_12_ is expected to measure the fraction of B_12_ bound to HC only.

### Genotyping

DNA was extracted from the subjects' baseline whole-blood samples, and were stored at −80°C with the use of a FlexiGene DNA kit (QIAGEN), as per the manufacturer's instructions. The genotyping of the single nucleotide polymorphism (SNP) *rs291466* in *HIBCH* was completed with the use of KASP assays at LGC Genomics. The quality of genotype data was assessed through the call rate of the SNP, which was > 98% and potential genotyping errors indicated by deviation from Hardy-Weinberg equilibrium (HWE).

### Statistical Analysis

All statistical analyses were performed using R software (44).

#### Exploratory Cross-Sectional and Longitudinal Correlations Between Demographic Variables and Four Biomarkers of B_12_ Status

Normal distribution of variables was assessed using the Shapiro-Wilk test, and parametric or non-parametric tests were used for those variables that significantly deviated from this distribution. Non-parametric Spearman correlations were used to assess the interrelationships between demographic factors (age and BMI) and B_12_ biomarkers, folate and cB_12_. Differences between trimesters were examined using analysis of variance (ANOVA) with integral *post-hoc* tests used for cB_12_ and BMI; and Friedman's test with Wilcoxon signed ranks tests for all other variables. All comparisons were two-sided; *p*-values were considered to deviate from the Null hypothesis using an α significance threshold of 0.05.

#### Effect of Folic Acid Supplementation on B_12_ Biomarkers Across Trimesters of Pregnancy

Previous studies have established that folic acid supplementation affects maternal serum folate status (45). The study participants in this cohort had low baseline tHcy measurements, potentially due to folic acid supplementation. We examined the effect of using folic acid and B_12_-containing multivitamin supplementation on all B_12_ biomarker levels in all trimesters of pregnancy using a simple means *t*-test. Supplementation data for our cohort is summarized in Supplementary Table 4.

#### Comparative Ranking of Vitamin B_12_ Biomarker Status

We created a comparative ranking system to assess and visualize both intra and inter individual variability of B_12_ status over the course of pregnancy. This method used a set of basic statistical rules, whereby higher concentrations of folate, sB_12_ and HoloTC, and “low” concentrations of MMA and tHcy were favored and assigned lower scores considered “more sufficient” starting at “1” and ranging to 50+, which was considered “more insufficient.” We then calculated the average of biomarker ranks for each individual at each trimester and plotted the rank scores over time.

### Genetic Association Analysis *HIBCH rs291466* With MMA and cB_12_ Within and Across All Trimesters of Pregnancy

We tested if *HIBCH rs291466* was in Hardy-Weinberg equilibrium (HWE) and then tested *HIBCH rs291466* for association with MMA concentrations and cB_12_ at T1, T2 and T3 of pregnancy) using the ***lm()*** function implemented in R. We did not use BMI and age as covariates in our analysis, although it has been established that they are associated with B_12_ status (22)—which was not the case in this cohort. To explore the effect of *HIBCH rs291466* during pregnancy on longitudinal changes in MMA and cB_12_ in the placebo group of the SPRINT cohort, we then calculated changes in MMA and cB_12_ from 12 to 35 weeks of gestation, and tested for genetic associations between the SNP and % change in MMA and % change in cB_12_ in R software, using the ***lm()*** function. We controlled for type I error rate by using the Bonferroni correction with the adjusted statistical threshold of significance: α' = α/*n*, whereby α is the nominal significance threshold for a single test (α = 0.05), and n equaling the total number of tests conducted (2 outcome variables (MMA & cB12) multipled by: 3 trimester-specific tests + two “longitudinal” tests for change (T1 to T2 & T1 to T3), yielding α' = 0.05/10 = 0.005.

## Results

### Summary Statistics of All Five Biomarkers at Each Trimester of Pregnancy

Data from a total of 95 healthy pregnant women were available for the study (Figure 1). Women had an average age of 31.1 y (±3.52). The mean BMI in T1 was 23.7 kg/m^2^ (±2.61), which increased to 24.9 kg/m^2^ (±2.61) in T2 and 27.8 kg/m^2^ (±2.89) in T3. Descriptive statistics of demographic and biochemical data for the cohort in each trimester are shown in Table 1. In the first trimester, the median sB_12_ concentration was 224 pmol/L (IQR 103) and 10% (*n* = 8) of women had concentrations below 148 pmol/L. Of these eight women, only one had an elevated MMA concentration. Concentrations of tHcy were generally low in this cohort, with a median of 4.6 μmol/L (IQR 1.3). Serum folate concentrations were above the deficiency cut-off for all participants in all trimesters. HoloTC concentrations ranged from 28 to >128 pmol/L, and 2% (*n* = 2) of women had a concentration below 32 pmol/L (46). MMA concentrations among our cohort of pregnant women ranged from 45 to 406 nmol/L, but only two women (2%) had concentrations considered elevated (Supplementary Table 1). Proportions of biomarkers outside of normal ranges in the T2 were similar to T1. This was with the exception of sB_12_, which increased to 12% (*n* = 11). Of these women, nine had all other biomarkers within normal range. In T3, only concentrations of tHcy were within normal range for every participant. Over a third of women (*n* = 32) had sB_12_ concentrations below 148 pmol/L and 72% (*n* = 23) of these had normal concentrations of all other biomarkers. The proportion of women with elevated MMA increased to 6% (*n* = 5); two of these had normal sB_12_ concentrations. In T3 8% (*n* = 7) of women had HoloTC concentrations below 32 pmol/L, all of whom had normal MMA concentrations.

There were significant changes between trimesters for all biomarkers of B_12_ status (HoloTC, tHcy, MMA, sB_12_) and folate (Table 1). No significant changes were observed for cB_12_ between T1 and T2. However, there was a significant decrease in mean cB_12_ in T3, with 5% (*n* = 5) of women having cB_12_ score below−0.5, the cB_12_ cut-off for “low” B_12_ status in non-pregnant population (37).

Histograms, showing the distribution of data points for each biomarker and cB_12_ across the 3 trimesters of pregnancy are given in Supplementary Figure 1. We used the Shapiro-Wilk test statistic (W) tested the null hypothesis that each biomarker is normally distributed at each trimester (Table 2). This demonstrated that none of the biomarker data is normally distributed at any time point, except cB_12_, which were normally distributed in trimesters 2 and 3.

**Table 2:** Correlation matrix of vitamin B_12_ status biomarkers, cB_12_ and [HoloTC: sB_12_] in trimesters 1, 2 and 3 of pregnancy.

		**Trimester 1**							**Trimester 2**							**Trimester 3**						
		**Folate**	**sB_**12**_**	**Holo-TC**	**HC**	**tHcy**	**MMA**	**cB_**12**_**	**Folate**	**sB_**12**_**	**Holo-TC**	**HC**	**tHcy**	**MMA**	**cB_**12**_**	**Folate**	**sB_**12**_**	**Holo-TC**	**HC**	**tHcy**	**MMA**	**cB_**12**_**
Trimester 1	Folate																					
	sB_12_	0.20																				
	Holo-TC	0.09	**0.64**																			
	HC	−0.12	−0.26	**0.52**																		
	tHcy	0.10	−0.11	−0.34	−0.27																	
	MMA	−0.04	−0.33	**−0.41**	−0.12	0.16																
	cB_12_	0.04	**0.73**	**0.79**	0.20	**−0.45**	**−0.65**															
Trimester 2	Folate	0.33	**0.39**	0.33	−0.02	−0.20	0.01	0.17														
	sB_12_	0.12	**0.82**	**0.47**	−0.26	−0.16	−0.15	**0.56**	**0.45**													
	Holo-TC	0.03	**0.42**	**0.82**	**0.55**	−0.36	−0.36	**0.57**	0.27	**0.38**												
	HC	−0.05	−0.15	**0.53**	**0.87**	−0.30	−0.32	0.32	−0.07	−0.29	**0.65**											
	tHcy	0.06	−0.20	**−0.41**	−0.31	**0.78**	0.15	−0.34	−0.37	−0.16	−0.33	−0.28										
	MMA	−0.11	−0.28	**−0.39**	−0.18	0.08	**0.82**	**−0.56**	−0.09	−0.17	−0.36	−0.27	0.11									
	cB_12_	0.05	**0.64**	**0.80**	0.31	**−0.47**	**−0.61**	**0.86**	**0.40**	**0.62**	**0.70**	0.38	**−0.54**	**−0.63**								
Trimester 3	Folate	0.16	0.22	0.17	−0.05	−0.20	−0.13	0.11	**0.56**	0.25	0.16	−0.05	−0.32	−0.14	0.27							
	sB_12_	0.14	**0.67**	**0.41**	−0.16	0.01	−0.15	**0.53**	0.30	**0.74**	0.13	−0.17	−0.12	−0.21	**0.53**	0.29						
	Holo-TC	0.09	**0.43**	**0.80**	**0.49**	−0.23	−0.31	**0.58**	0.25	0.33	**0.71**	**0.55**	−0.27	−0.31	**0.65**	0.22	0.46					
	HC	−0.02	−0.15	**0.44**	**0.70**	−0.32	−0.16	0.19	−0.06	−0.29	**0.49**	**0.77**	−0.24	−0.11	0.22	−0.08	−0.35	**0.60**				
	tHcy	−0.14	−0.19	−0.33	−0.13	**0.63**	0.03	−0.22	**−0.47**	−0.14	−0.28	−0.15	**0.63**	−0.02	−0.32	−0.41	0.00	−0.24	−0.21			
	MMA	−0.07	−0.15	−0.21	−0.07	0.17	**0.71**	**−0.47**	−0.02	−0.09	−0.20	−0.17	0.13	**0.71**	**−0.41**	−0.13	−0.11	−0.17	−0.08	−0.01		
	cB_12_	0.08	**0.54**	**0.66**	0.16	−0.36	**−0.49**	**0.72**	**0.39**	**0.48**	**0.54**	0.24	**−0.38**	**−0.47**	**0.73**	0.36	**0.57**	**0.72**	0.21	**−0.45**	**−0.58**	

*P-values significant at Bonferroni-adjusted significance levels of 0.05 or less are presented in Bold*.

*P-values nominally significant at p < 0.05 are presented in yellow*.

Given this lack of normal distribution, the standard method of defining outliers (and therefore also defining those who could be insufficient) using standard deviation from the mean is difficult. Instead, the 95th [for MMA / tHcy] 5th centile [for serum folate, B_12_ & HoloTC] was used to suggest potential trimester-specific reference ranges for vitamin B_12_ biomarkers (Supplementary Table 8).

### Effect of Use of Folic Acid Supplementation on B_12_ Biomarkers Across Pregnancy

There were a total of *n* = 113 women taking folic acid and multivitamin supplements during the first trimester of pregnancy. However, this number decreased to *n* = 29 and 5 in the second and third trimesters (Supplementary Table 4). We examine the effects of using (i) folic acid supplements (400 mcg) on tHcy levels of women in T1, and (ii) active B_12_-containing multivitamins on B_12_ levels across the 3 trimesters in the individuals included in the study. There were no significant differences in mean tHcy levels between women taking folic acid supplements and those taking B_12_-containing multivitamins (*P* > 0.05). Accordingly, there were no significant differences in folate levels between the two groups, as both were subjected to folic acid intake. To identify the effects of B_12_-containing multivitamins on B_12_ levels in our cohort, we performed Wilcoxon-signed ranks tests to identify significant differences in B_12_ means between women taking folic acid supplements (without B_12_) and women taking folic acid and B_12_-containing multivitamins. There were no significant differences in sB_12_ (Supplementary Table 5).

### Longitudinal Trends of Biomarkers of Vitamin B_12_ Status in Pregnancy

**Figure 2:**
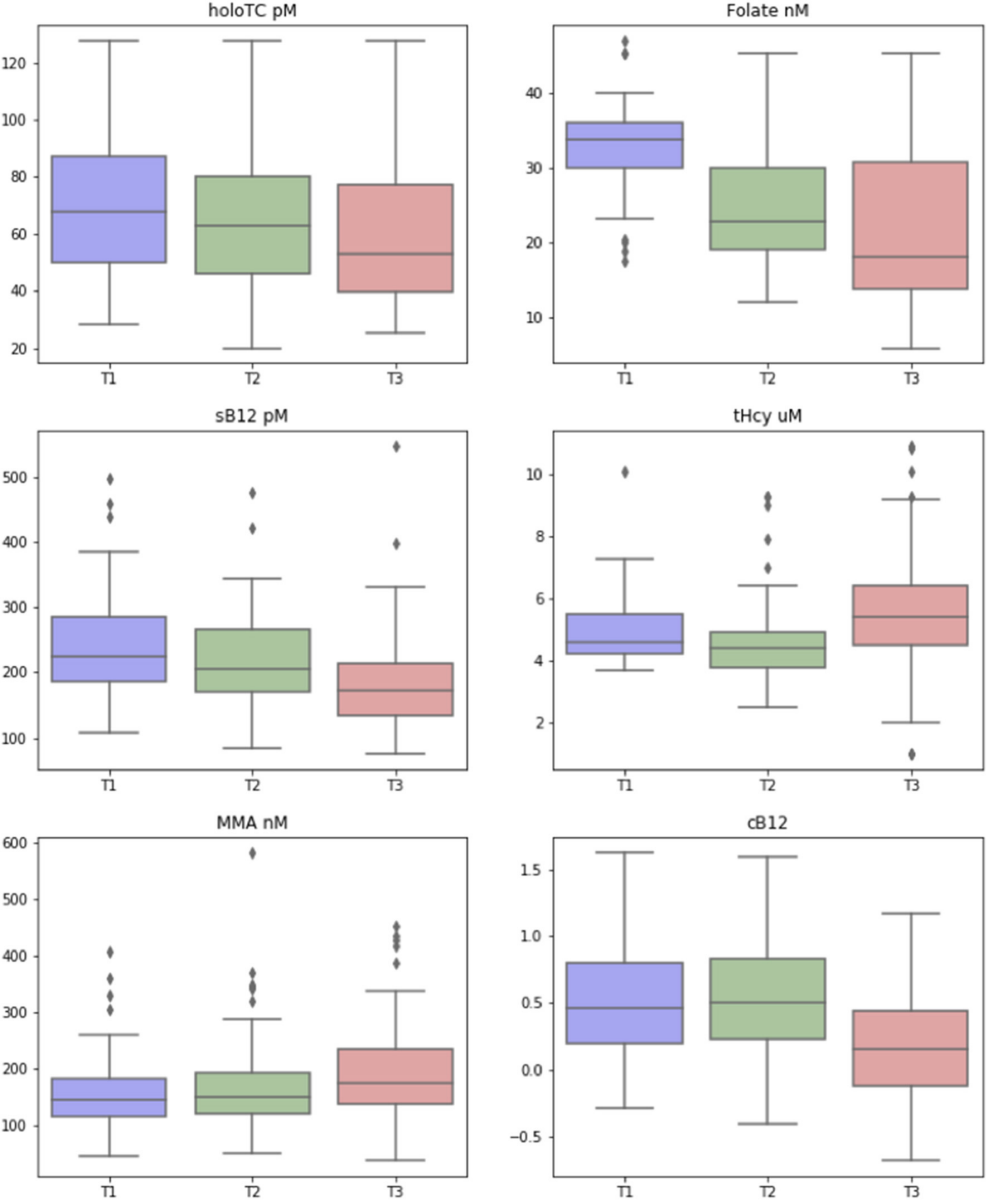
Change in biomarkers of vitamin B12 status over the three trimesters of pregnancy. Box-and-whisker plots, showing how the biomarkers of vitamin B_12_ [including transcobalamin (Holo TC, pmol/L), serum folate (Folate, nmol/L), serum B_12_ (pmol/L), homocysteine (tHCy, micromol/L), methylmalonic acid (MMA, nmol/L), and combined B_12_ (cB_12_)], changed across the three trimesters (T1, T2, T3) of pregnancy. The colored box represents the inter-quartile range (with median as the horizontal line), and the whiskers show the entire range of values considered to be within the homogeneous distribution, while diamonds represent the assumed outliers according to Tukey's fences (median ± 2 × interquartile distance).

We performed longitudinal analysis to examine how biomarker data and cB_12_ score changed over the three trimesters of pregnancy. These changes are represented in Figure 2. We used 2 sided, one sample *t*-tests to test whether the changes demonstrated were statistically significant. This showed that there was indeed a significant change between all of the biomarkers over time, with the exception of cB_12_ score between the first and second trimesters. Results (including 95% confidence intervals for these changes calculated using bootstrapping due to lack of normal distribution) are presented (Supplementary Figure 2). Median concentrations of sB_12_ (−23%) and holoTC (−22%) decreased and tHcy (18%) and MMA (22%) increased significantly from trimester 1 to 3 (*p* < 0.001). Reductions in serum folate were also observed between each trimester, most notably between the first (median 33.7 nmol/L, IQR 6.2) and second trimesters (22.9 nmol/L, IQR 11.2) largely due to mothers stopping their use of folic acid supplements. The most notable change was the dramatic decrease in the cB_12_ score, where the values were identical between trimesters 1 and 2 but decreased by 66% in trimesters 3 (*p* < 0.001). Interestingly, the concentrations in those women with the lowest baseline (i.e., Trimester 1) values of sB_12_ showed no significant differences (*p* > 0.05) between any of the trimesters. In the case of MMA, however, the trend of increase across T1-T3 was more significant in those women within the lower quartile of MMA at baseline (i.e., T1).

### Trimester 1 HoloTC Concentrations Are Correlated With All Static and Functional Markers of Vitamin B_12_ Status Throughout Pregnancy

In general, we observed that all markers in T1 were highly and significantly correlated with themselves in T2 and T3 (*R* ranged from 0.63 to 0.86 | *P* < 0.001). There were significant correlations between sB_12_ in T1 and HoloTC, MMA concentrations in T1-T3 (*r* = 0.6, *P* < 0.05). Serum B_12_ concentrations were also highly, consistently and significantly correlated with cB_12_ across the three trimesters; *R* was 0.74, 0.64, and 0.54 in T1 through to T3 (*P* < 0.001), However, there were low and insignificant correlations between sB_12_ and tHcy in all the 3 trimesters and between sB_12_ and HC in T2 and T3 (R < −0.15 | *p* = 0.15) although we did observe a weak correlation in T1 (*R* = −0.26 | *P* = 0.001).

HoloTC in T1 was highly and significantly correlated with all static and functional markers of vitamin B_12_ status throughout pregnancy although the correlations, except for holoTC in T2 and T3 where *R* remained >0.8 | *P* < 0.001, all tended to become weaker as the pregnancy progressed (Table 2). This highlights potentially a reduction in the predictive capability of HoloTC in trimester 1 for functional indicators of B_12_ status (MMA and tHcy) in the later stages of pregnancy (T2 & T3) where there is a significant increase in demand from both the mother and the fetus. Note that this was also true if we used HoloTC measurments in T2 and T3; i.e., measuring HoloTC across all 3 trimesters does not mitigate the lack of predictability of tHcy and MMA by HoloTC in T1. However, HoloTC was highly correlated with cB_12_ scores in all 3 trimester of pregnancy with R values of 0.7-0.8 across T1-T3 (p<0.001). In general, both MMA and tHcy were both poorly predicted (correlated) and predictors of each other and the other B_12_ biomarkers (sB_12_, HoloTC, and HC) although they were both highly correlated with cB_12_ in all 3 trimesters of pregnancy (Table 2). Lastly, it is important to highlight that folate concentrations were not correlated with any of the markers of B12 status in T1 through to T3.

### cB_12_ Significantly Correlates With Serum Vitamin B_12_, HoloTC, tHcy and MMA at Each Trimester of Pregnancy

cB_12_ (T1) was highly and significantly correlated with cB_12_ (T2) (*r* = 0.86, *P* < 0.001) and cB_12_ (T3) (*r* = 0.72, *P* < 0.001) as well as with all other markers of B_12_ status across all 3 trimesters of pregnancy although it was generally weakly correlated with tHcy in T2 and T3 (Table 2). These results suggest that cB_12_ in T1 consistently captured B_12_ status biomarkers MMA, HoloTC, sB_12_ in T1 through to T3 of pregnancy.

### Behavioral Consistency of Individual-Level B_12_ Biomarkers During Pregnancy

**Figure 3:**
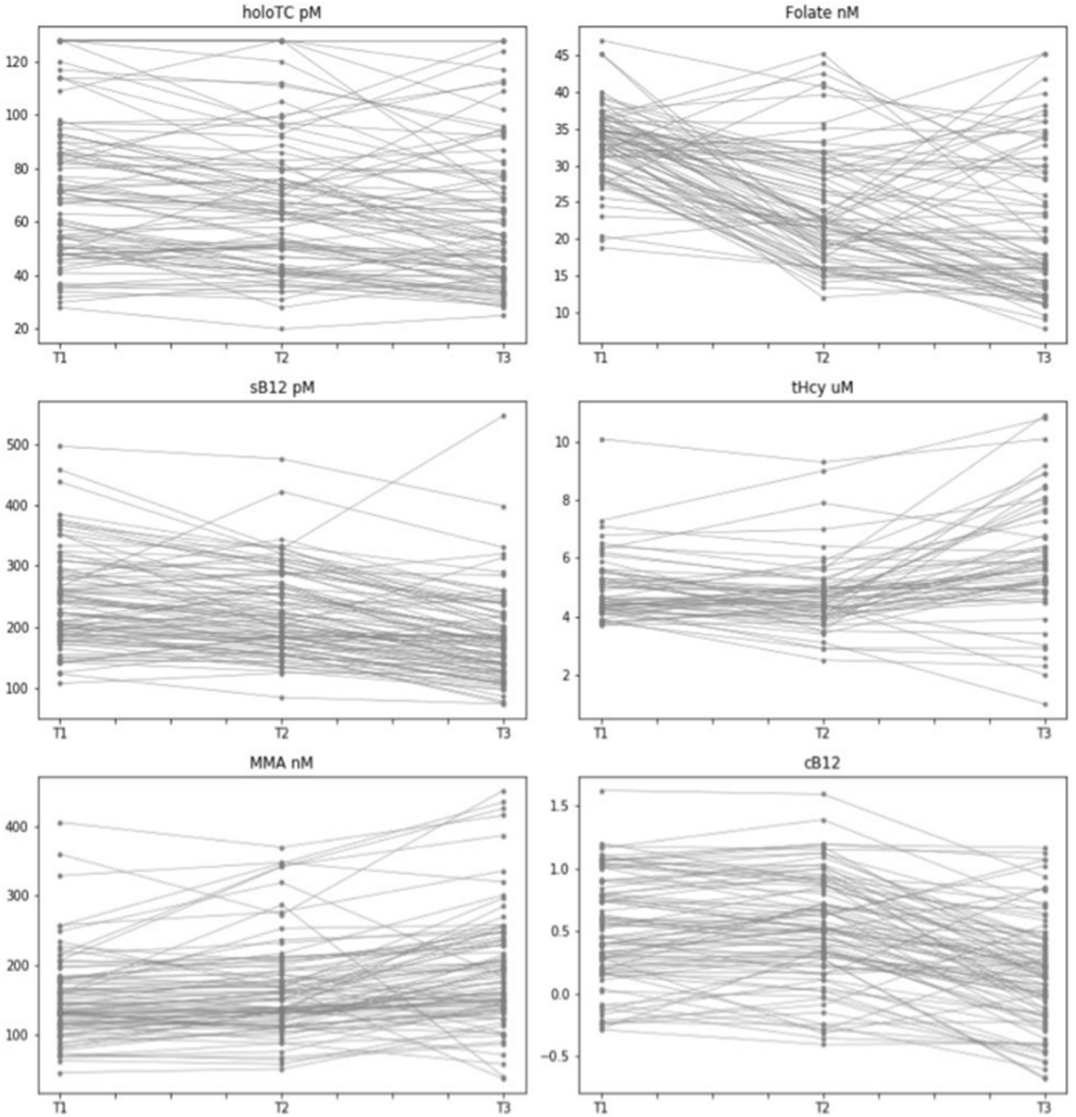
Intra- and Inter-individual variability of vitamin B_12_ biomarkers measured across the three trimesters of pregnancy. Graphical representation of pregnant women with data points for biomarkers of vitamin B_12_ status across the three trimesters of pregnancy (T1, T2, T3), indicating consistency of behavior for each biomarker for each individual of this dataset. Biomarkers include Holo TC (pmol/L), serum folate (nmol/L), serum B_12_ (pmol/L), tHcy (μmol/L), MMA (nmol/L), and cB_12_.

In order to get an indication of the intra-individual variability and visualize the consistency in behavior of each biomarker at an individual level we plotted values of B_12_ biomarkers for each individual across the three trimesters of pregnancy (Figure 3). This would also allow us a to gain a visual comparison of biomarker behavior to other (pregnant) women at each trimester of pregnancy. Our visual data suggests that whilst there is a relatively significant amount of intra-individual variability for all biomarkers, in particular for HoloTC and cB_12_, there are group-level upward trends in tHcy and MMA concentrations and downward trends in folate and sB_12._ We observed no distinct trends for HoloTC and cB_12_ (Figure 3).

### Average Rank Across Vitamin B_12_ Biomarkers Over Time

**Figure 4:**
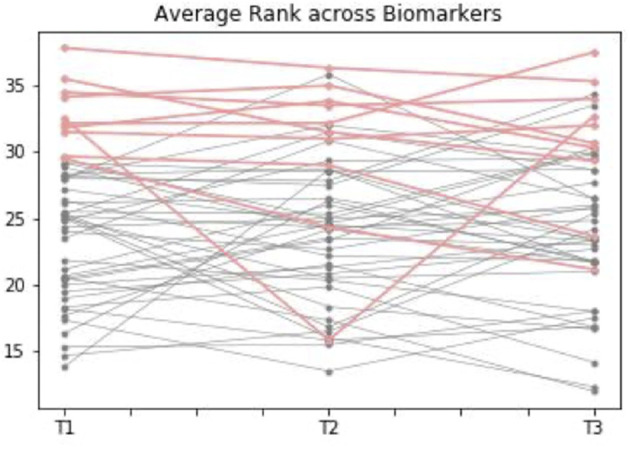
Average comparative rank of vitamin B_12_ biomarker status during the three trimesters of pregnancy. This graph shows how individuals with complete data points available for biomarkers of vitamin B_12_ [including transcobalamin (holoTC), serum folate (Folate), serum B_12_, homocysteine (tHCy), methylmalonic acid (MMA), and cB_12_] rank across the three trimesters. Ranking was calculated according to a set of statistical rules: it was considered “better” to have high folate, sB12, cB12 and holoTC, but low MMA / tHcy, where 1 is “more sufficient” and 50+ is “more insufficient.” The average of biomarker ranks for each individual was calculated at each trimester. When the “most insufficient” 10 individuals (marked in the graph as red) were plotted in Trimester 1 [T1], 7 of these remained in the lowest group at Trimester 2 [T2] and Trimester 3 [T3], suggesting there might be a subgroup at risk of insufficiency based on first trimester measurements.

We created an *ad-hoc* system using a set of simple statistical rules to examine how groups of individuals performed over the course of pregnancy. This process helped to identify if there was a subset of women at higher risk of vitamin B_12_ deficiency, who could potentially be identified from the first trimester. When the “most insufficient” 10 individuals (marked in Figure 4 as red) were plotted in T1, 7 of these remained in the lowest group at T2 and T3, showing a degree of consistency in the likelihood of deficiency across all the trimesters, and suggesting that it might be this subgroup most in need of further investigation or follow-up based on their first trimester measurements.

### Prediction of Women at Risk of Vitamin B_12_ Deficiency Over the Course of Pregnancy

**Figure 5:**
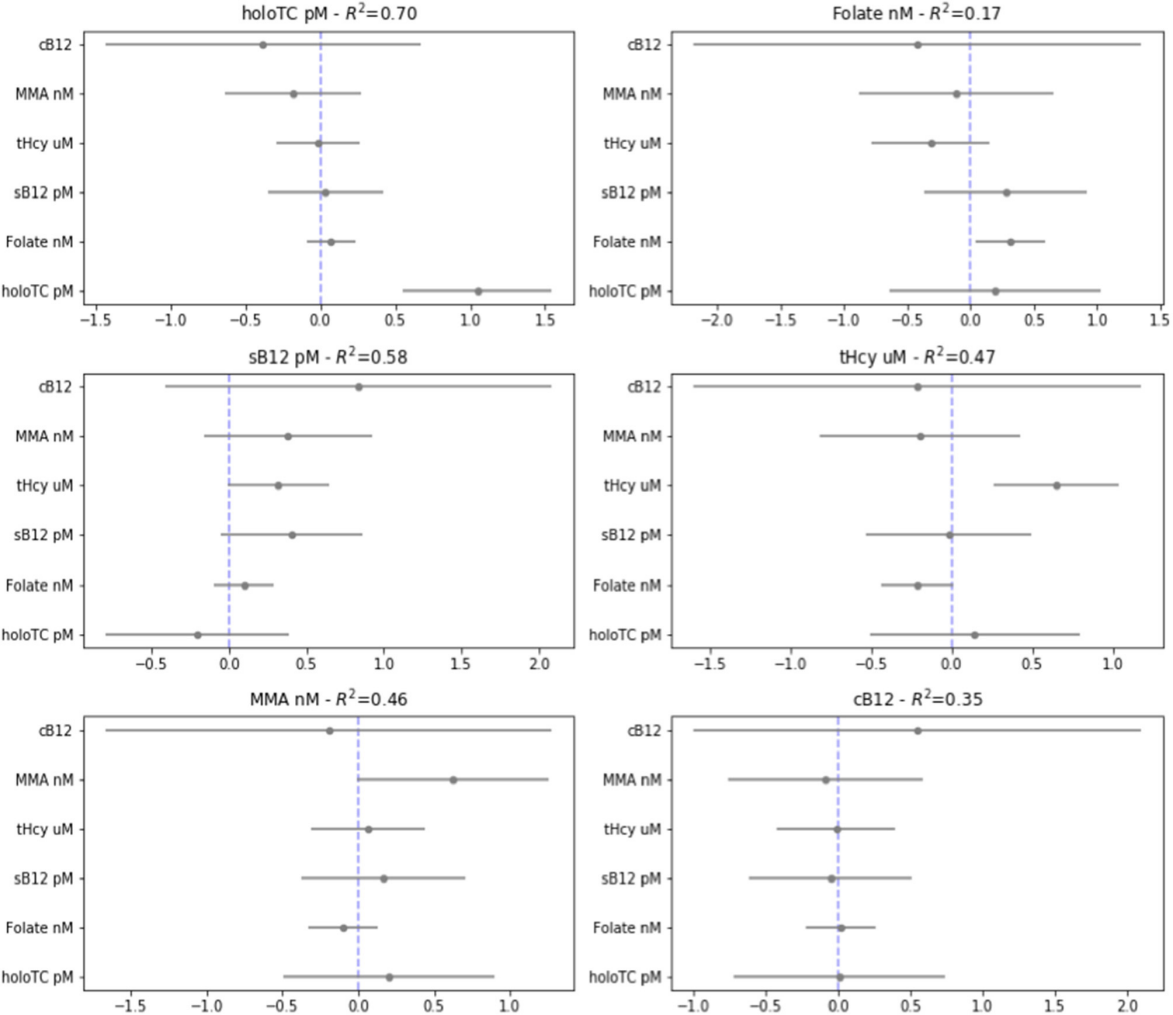
Regression analysis from the third to the first trimester. Visualization of multiple linear regression analysis, showing coefficient and standard errors, examining whether each biomarker [transcobalamin (Holo TC, pmol/L), serum folate (Folate, nmol/L), serum B_12_ (pmol/L), homocysteine (tHCy, micromol/L), methylmalonic acid (MMA, nmol/L), plus score of combined B12 (cB_12_)] in the third trimester of normal pregnancy [T3] can be significantly predicted by the same or the other baseline biomarkers measured in first trimester of pregnancy [T1] (given on the y-axis). This was calculated using a standardized outcome variables for linear regression, with 95% confidence intervals on the coefficients.

We used multivariate regression analysis to examine if any of the biomarkers measured in the third trimester (a representation of the end of pregnancy) could have been significantly and reliably predicted by any individual biomarkers (or combination thereof) measured in the first (illustrated in Figure 5) and second (illustrated in Supplementary Figure 3) trimesters. All inputs for the regression analysis were first normalized, to enable coefficient comparisons.

To our surprise, the only statistically significant predictor of biomarker values in T3 was in general that same biomarker measured earlier in time; i.e., in T1 or T2; the exception was of folate in the second trimester, which was a predictor of homocysteine in the third trimester. Folate, holoTC, and tHcy could significantly predict themselves, but no other biomarkers, between trimesters three and one. Notably, MMA and sB_12_ did not reach statistically significant predictive value even for itself across this time period. The cB_12_ score did not significantly predict itself or any of the other biomarkers between trimesters. Please note, however, there are limitations of these regression analysis models, and attention should be paid to the R squared values, which with perhaps the exception of holoTC are generally relatively low in both analyses (see Supplementary Figure 4 for potential limitations of this analysis).

### Genetic Association Between *HIBCH rs291466*, MMA and cB_12_

Association between HIBCH rs291466 and MMA and cB_12_ at T1, T2, T3 of pregnancy. The *HIBCH rs291466* SNP did not deviate from Hardy- Weinberg equilibrium (*P* > 0.05) (Supplementary Table 3). Characteristics of *HIBCH rs291466* including the position, minor allele frequency, and *P*-value for Hardy-Weinberg equilibrium are presented in Supplementary Table 3. Results of the association between the SNP and cB_12_ and MMA status at T1, T2 and T3 are shown in Table 3. Our results show that *HIBCH rs291466* was significantly associated with MMA concentrations at 12-week gestation (T1 baseline) (*P* = 8.46e-05), T2 (*P* = 0.0001), and T3 (*P* = 0.03) explaining 16, 14, and 4% of the variance in MMA. The direction of the association was negative, as expected, suggesting that the additive effect of the A allele contributes to a reduction in MMA concentrations. Table 3 also shows that *HIBCH rs291466* was associated with cB_12_ at T1 baseline (*P* = 0.0005), T2 (*P* = 0.003), and T3 (*P* = 0.03) explaining 12, 8, and 5% of the variance in cB_12_. We would like to highlight that these results show that that the predictive ability of the T3 MMA and cB_12_ for the *HIBCH rs291466* are not significant (data not shown).

**Table 3:** Association between the *HIBCH rs291466* variant with cB_12_ and MMA at T1, T2, and T3 of pregnancy^a^.

		**Geometric mean (95% CI)**					
**Biomarker**	**Subjects *n***	**AA**	**AG**	**GG**	**β ±SE**	***R^**2**^***	***P*-value**
MMA T1	90	102.30	135.91	174.39	−35.81 ± 8.68	0.16	8.5e-05^*^
MMA T2	94	113.57	141.39	187.33	−42.69 ± 10.64	0.14	1e-0.5^*^
MMA T3	89	151.54	158.41	191.55	−25.81 ± 12.15	0.04	3.6e-02
cB_12_ T1	91	0.70	0.46	0.29	0.21 ± 0.05	0.12	1e-0.5^*^
cB_12_ T2	93	0.69	0.45	0.30	0.17 ± 0.05	0.08	3e-0.3
cB_12_ T3	91	0.23	0.10	0.00	0.13 ± 0.06	0.05	3.2e-02

a *All genetic analyses were carried out using the linear regression **lm()** function within the R software to test the effect of the exposure variable (rs291466 genotype) on the outcome variables (MMA and cB_12_ T1, T2, T3)*.

* *Indicates considered significant P-values after Bonferroni correction (P < 0.0005)*.

*cB_12_, combined indicator of B_12_ status; HoloTC, holotranscobalamin; sB_12_, serum B_12_; tHcy, total homocysteine; MMA, methylmalonic acid*.

Finally, having showed that MMA concentrations significantly increased between T1 and T3 (from 143 nmol/L in T1 to 174 nmol/L in T3, *P* < 0.001), and cB_12_ significantly decreased from 0.5 to 0.17 (*P* < 0.001) (Table 1), our results show that the *HIBCH rs291466* was not associated with T1-T3 changes in MMA or cB_12_ in this study (Supplementary Table 7).

## Discussion

Antenatal screening of vitamin B_12_ status in the UK is currently piecemeal (47). This is in light of the overwhelming evidence linking vitamin B_12_ insufficiency/deficiency during pregnancy with a number of adverse fetal and obstetric complications, including neural tube defects (NTDs), recurrent pregnancy loss, infertility and preterm birth (5, 13, 14). There are also significant gaps in our understanding around the expected or “normal” behavior of static and functional biomarkers of vitamin B_12_ status, including the wellness score or cB_12_, over the course of normal pregnancy. Understanding the complexity of maternal—as well as placental and fetal—adaptations to pregnancy and finding biomarkers with sufficient sensitivity and specificity to reflect such adaptations during the course of a healthy pregnancy, is therefore a major research focus.

This study aimed to further develop our understanding of how static—serum B_12_, folate, HoloTC—and functional—tHcy, MMA—biomarkers of vitamin B_12_ status behave over the course of healthy pregnancy, and if there is any reliable predictive value of such biomarkers to help identify women at the initial stages of pregnancy who may be at elevated risk of vitamin B_12_ insufficiency/deficiency.

### Summary of Results

Our findings suggest that there was a statistically significant change for all of the biomarkers over the course of three trimesters of pregnancy, with the exception of cB_12_ which remains stable during the first two trimesters of pregnancy, but declines significantly in the third trimester. Our findings, which are in line with previous studies—strongly support the importance of creating trimester-specific B_12_ reference ranges as opposed to a unified “reference range of pregnancy.” Secondly, our results show a discordance between particular individuals and cohort trajectories of B_12_ status over the course of pregnancy. It is interesting to note that while many mothers did tend to follow the cohort patterns of biomarker behavior, in all cases there were specific strata of women who varied widely between T1 through to T3 and did not conform to the group-level changes (Figure 3). This is perhaps in part as a result of alterations in renal function, change in dietary pattern, genetic variation, haemodilution or any number of as yet undefined physiological shifts. Nevertheless, this highlights the need for developing a reliable diagnostic in order to identify those subgroups of women who may be at elevated risk of B_12_ deficiency at an early point in their pregnancy, so that appropriate intervention and follow-up may be instigated as soon as possible. We purport this to be a key public health priority. To this end, we carried out exploratory correlation analysis, an *ad-hoc* ranking system and multivariate regression analyses to identify any biomarkers or combinations thereof with reliable predictive value to help identify women who may be at elevated risk of B_12_ insufficiency/deficiency over the course of pregnancy. Our correlation analysis showed that sB_12_, HoloTC and cB_12_ are all correlated with the temporal behaviors of all the B_12_ biomarkers across T1-T3 of pregnancy. Yet, HoloTC might appear a consistent diagnostic tool of B_12_ status in pregnancy, if analysis of our cohort with B_12_-adequate participants can be extrapolated to a truly B_12_-deficient population of pregnant women. Our *ad-hoc* ranking system showed that of the 10 individuals deemed ‘most insufficient' in trimester 1, 7 were also in the lowest group in the next two trimesters. This suggests a relatively high degree of consistency between those with the lowest vitamin B_12_ status ranking in the first trimester, and their relative status at the end of pregnancy. It is these women who may benefit most from further investigation and follow-up. Subsequently, multivariate regression analysis was performed between the biomarkers measured in T3 - a representation of B_12_ status toward the end of pregnancy - and T1 and T2 to see if any biomarker, or combination of markers, could reliably predict and identify these women whom are putatively at elevated risk of vitamin B_12_ insufficiency/deficiency at the latter stages of pregnancy. Our results showed that the only statistically significant predictor of future biomarker values was the same biomarker measured earlier in time. This was the case for all biomarkers between trimester three and two and for folate, holoTC, and tHcy between trimesters three and one. Interestingly, cB_12_ did not significantly predict itself between any of the trimesters. This analysis provides an important null finding – that most individual biomarkers, including cB_12_, are not reliably predictive of anything other than themselves – at least within the specific context of this relatively small cohort of healthy, pregnant individuals. Despite the statistical significance of the multivariate analyses, it is important to consider that the R-squared values were relatively low for almost all data points, with perhaps the exception of HoloTC. Larger cohort studies are therefore required to improve the accuracy of this analysis.

Finally, we also replicated significant associations between the known *HIBCH* rs291466 genetic variant with MMA and cB_12_ across the 3 trimesters of pregnancy although interestingly the *HIBCH* variant had no significant effect on the longitudinal changes in MMA concentrations and cB_12_ throughout pregnancy.

### Are Trimester-Specific Reference Ranges Comparable to Those From the General Population?

Comparing biomarker values in pregnancy with those of non-pregnant reference ranges is useful in terms of establishing differences and similarities to a more established baseline. However, directly comparing these two population groups is inevitably challenging, as values that fall within normal ranges outside of pregnancy may not represent optimization of risk reduction during pregnancy and embryogenesis. It is also important to remember that non-pregnant reference ranges themselves differ between laboratories (27, 47, 48).

However, with these limitations firmly in mind, it is interesting to note that the mean of the biomarker values in our study fell within the broad reference ranges outlined by the British Society for Hematology (BSH) guidelines (and within the range of “B_12_ adequacy” for score of cB_12_) for non-pregnant individuals, at all time points across pregnancy, except for sB_12_ (Supplementary Table 8). This aligns with the findings of Morkbak et al. (11), who noted that HoloTC concentrations in pregnancy were comparable to the concentrations seen in healthy, non-pregnant women, as well as those of Murphy et al. (30), who found the MMA concentrations they measured did not indicate deficiency at any time during pregnancy. The reference ranges suggested for serum B_12_ (<141 and <134 pmol/L), HoloTC (<35.5 and <35.1 pmol/L) and MMA (>258 and >342 pmol/L) in the first two trimesters (Supplementary Table 8) are comparable with reference ranges for these biomarkers that have been identified by Schroder et al. (36): sB_12_ (<89.9, 84 pmol/L), HoloTC (<29.5, <26.0 pmol/L) and MMA (>371, >374 nmol/L). Further work is required to build on these initial findings.

Our results highlighted a decrease in sB_12_ across trimesters 1 through to 3 of pregnancy, in line with findings from previous studies (3, 27–29). This could be deemed as a natural consequence of progression in pregnancy and increased demand as it has been previously shown that sB_12_ remains constant throughout pregnancy in Nigerian women with adequate B_12_ intakes (48).

MMA concentrations increased slightly but significantly throughout the three trimesters although it remains unclear what the exact etiology of this increase is; i.e., whether it is due to increased cellular energy needs, and subsequent upregulation of bioenergetic pathways, including the mitochondrial conversion of methylmalonyl-CoA to succinyl-CoA catalyzed by the B_12_-dependent MUT enzyme, or due to deteriorating B_12_ status (30, 47). Studies have shown that lower pre-conceptional HoloTC concentrations are associated with higher MMA concentrations in pregnancy (30). However, we did not have access to pre-conceptional data in order to replicate these findings. Data on HoloTC from the first trimester in our cohort shows that those in the lowest tertile of HoloTC exhibit higher MMA concentrations. Despite these changes, 95th centile cut-offs for MMA remained within “normal” ranges until the third trimester, when it slightly elevated above the non-pregnant reference range cut-off (here defined as >350 nmol/L, although again, it should be noted that this is not a universally defined consensus).

In the case of tHcy (3, 31), we observed a non-significant drop in tHcy between T1 and T2 followed by a highly significant increase in T3 although tHcy concentrations were generally low in this cohort probably as a result of the prevalent use of folate containing supplements in T1 of pregnancy. Thus, in line with previous studies (3, 28) our study shows that tHcy is a non-specific indicator of B_12_ status in pregnancy, maybe in small part due to genetic factors (14, 49) and status of other nutrients, including methionine, betaine, B_2_ and B_6_ (24, 26, 50), but largely due to the effects of haemodilution, increased GFR, hormonal influences.

Finally, in contrast to findings from our study, where mean/median values for HoloTC decreased between T1 through to T3 (although the wide, and over-lapping confidence intervals should be noted), other studies have found HoloTC to remain relatively stable throughout pregnancy (11, 29, 51) with one study by Greibe et al. noting a small increase in HoloTC from weeks 24–36 or T2–T3 (29). Our findings align with those of Koebnick et al., that suggested that a reduction in HoloTC concentrations toward the end of pregnancy may be a product of altered protein binding capacity rather than a representation of B_12_ inadequacy (3). Previously, Murphy et al. had shown a decrease in HoloTC at 8 weeks' gestation from conception (30) suggesting that noteworthy changes in HoloTC occurred in T1 of pregnancy. Our findings showed consistently high correlations between HoloTC and sB_12_ concentrations in all three trimesters of pregnancy, in line with a previous study that highlighted increases in HoloTC with higher B_12_ intakes (52). Although we did not have this data it is important to highlight that post-partum increases in HoloTC have also been reported previously (11, 51) but the mechanisms underpinning these trends remain unknown. Additionally, HoloTC showed consistent, moderate (negative) correlations with both MMA and tHcy (indicators of functional B_12_ status) throughout the three trimesters of pregnancy. Murphy et al. has also shown that HoloTC at 32 wk gestation was negatively correlated with cord MMA concentrations, and that women with lower HoloTC at preconception had greater increases in MMA at 32 wk, suggesting that initial HoloTC measurements are predictive of increased MMA concentrations at later stages of pregnancy in both the mother and the infant. We would also highlight that the high heritability of HoloTC (*h*^2^= ~70%) (38) and HC [i.e., [HoloTC:sB_12_]] are in line with our findings of its biological stability throughout pregnancy and support its use as a useful diagnostic of functional B_12_ status during pregnancy.

### Most Suitable Markers of Vitamin B_12_ Status During Pregnancy

One of the principle objectives of our study was to explore the behavior and putative diagnostic value of the composite score cB_12_ in identifying those at risk of B_12_ deficiency throughout pregnancy. Our results showed that cB_12_ measured at T1 was significantly correlated with cB_12_ measured at T2 and T3 as well as with sB_12_, HoloTC and MMA, but not tHcy in all trimesters of pregnancy. Results from our comparative ranking system for B_12_ status also suggested that combined scores of the five biomarkers of B_12_ status—which would be similar to cB_12_–for individuals that ranked “most insufficient” at T1 were consistent with “most insufficient” scores at T2 and T3 (additional data shown in Supplementary Table 9). However, cB_12_ performed very poorly in our multivariate regression analysis where it didn't even significantly predict itself between any of the trimesters. This may be explained by the absence of vitamin B_12_ deficiency in our cohort of pregnant women: none of the participants were B_12_-deficient at the start of the study, and only 5 subjects at T3 fell below cB_12_ = −0.5 (categorized as “decreased B_12_”). Because cB_12_ was primarily designed to overcome fluctuations in B_12_ status that are not “true,” these results are expected in the context of this cohort. Conducting the same analysis in a B_12_–deplete population—i.e., a cohort pregnant women or with low meat intake/vegan may further elucidate the predictive/prognostic value of cB_12_. Furthermore, calculation of cB_12_ as part of routine antenatal practice would be prohibitively costly, as it requires the simultaneous measurement of several biomarkers; Although, it may be feasible to utilize cB_12_ derivatives, consisting of two or more biomarkers, as indicators of B_12_ status throughout pregnancy in place of cB_12_.

Taken together, our findings do suggest that cB_12_ may be a poor and expensive diagnostic tool for identifying B_12_ deficiency in pregnancy. Closer inspection of Table 2 shows that within each trimester, the strongest correlates for cB_12_ were HoloTC and MMA. While sB_12_ remains a first-line test for B_12_ status across the lifecourse, MMA and HoloTC, have been previously established as specific biomarkers for second-line testing of B_12_ status in the literature (50, 53) and shown to be the most reliable biomarkers of B_12_ status (37) in the general population. Results from our study reinforces the merits of a derived score based on the simultaneous parametrization of HoloTC and MMA together. From our data, unlike cB_12_, adjustments for age or folate status may be also unnecessary during pregnancy. However, the prospect that fewer analytes may be required to achieve a proxy for B_12_ status is intriguing and warrants further investigation.

### *HIBCH* Genotype Data Was Redundant in Terms of Diagnostic and Prognostic Value

Another major objective of our study was to evaluate the potential utility of *HIBCH rs291466*—which accounts for all the heritable variation in MMA and is strongly associated with variability in cB_12_–in diagnosing risk of vitamin B_12_ insufficiency/deficiency during pregnancy. Our results confirmed the association between *HIBCH rs291466* with both MMA and cB_12_ throughout all three trimesters of pregnancy although with diminishing *R*^2^ as you move from T1 to T3. The explained variance in MMA and cB_12_ was highest in the first trimester, 16 and 12% respectively and lowest in T3 (4 and 5% respectively) and in fact insignificant failing Bonferroni correction. Two points warrant discussion. First, the relationship between the *HIBCH rs291466* and MMA or cB_12_ is very similar in the first trimesters of pregnancy and to that observed in the general population (38) but this relationship deteriorates significantly as the pregnancy progresses through to trimesters 2 and 3 (Table 3). Second, the *HIBCH rs291466* SNP does not explain the significant change in MMA concentrations or cB_12_ observed through the pregnancy (Supplementary Table 7). The observed deterioration in the relationship between *HIBCH rs291466* SNP and MMA may be in large part related to added environmental sources of variability (as shown by the increase in IQR) in MMA. Finally, although the notion of utilizing the *HIBCH rs291466* as a biomarker for predicting which mothers are likely to be at elevated risk of B_12_ deficiency in pregnancy seems attractive, our results suggest that the SNP is not likely to be useful in this regard.

### Strengths and Limitations of the Study

This is the first study to use the context of pregnancy to observationally investigate (i) the utility of the composite score cB_12_ as a potential diagnostic and prognostic tool in assessing and predicting B_12_ status during pregnancy. Furthermore, the inherent cross-sectional and longitudinal aspects of the study design allowed us to utilize a variety of data analysis techniques that offer a range of insights into the inter-relationships between cB_12_, its constituent static/functional markers as well as *HIBCH rs291466*, across the three trimesters of a healthy.

There are, however, a number of limitations to this study that warrant consideration. Firstly, given the moderate sample size findings from our study need to be replicated in larger cohorts and validated across different ethnic groups. It is important to highlight that the power of our study was diminished as a result of missing data/samples. As a result, although we used a Bonferroni correction to decrease the likelihood of type I errors, the likelihood of type II errors is increased, so that truly significant results may have been deemed non-significant (54). Secondly, the first blood draw in the SPRINT study was at around 12 weeks' gestation—i.e., the end of the first trimester of pregnancy. Studies have previously shown that HoloTC and sB_12_ levels decrease from preconception to 8 weeks' gestation (30). Thus, significantly larger studies, including the use of different assay techniques, modalities and timepoints, are necessary to more accurately define the reference ranges of vitamin B_12_ biomarkers in pregnancy. Thirdly, information on the use of supplementation pre-conceptionally, general dietary patterns (including consumption of animal produce or not), renal function, haemodilution, microbiome composition and various other physiological changes of pregnancy (55) were not available and thus not included in the analysis, but may be important confounding factors that require further investigation. Finally, lack of any post-partum data on both the mother and newborn is a major shortcoming of the study.

### Guiding the Formulation of Precision Public Health Policy in Pregnancy

Perhaps the most pressing opportunity for further research is to establish a set of vitamin B_12_ biomarker reference ranges for both the pregnant, trimester-specific, and non-pregnant populations across different ethnic, vegan/vegetarian groups as well as those with pregnancy-related complications (pre-eclampsia or gestational diabetes) or pre-existing medical conditions. Only when these ranges are defined nationally can practitioners order and interpret test results with confidence, and thus stratify risk of (or indeed, treat) insufficiency/deficiency in their “patients.”

Further work is also needed to identify reliable ways of predicting the course of vitamin B_12_ status by testing at the beginning of pregnancy, or even preconception. As shown, there appears to be a subset of women (~10%) who suffer from low vitamin B_12_ status in the first trimester, which continues consistently into the second and third trimesters. Results from our multivariate regression analyses did not identify cB_12_ to have a statistically significant predictive value. Although HoloTC alone or in combination with MMA did show promise in this regard, our data suggests that while there is likely to be a cohort of women who are at elevated risk of vitamin B_12_ deficiency right from the start of their pregnancy, we are not yet able to accurately and reliably identify who they may be.

Finally, once reference ranges have been defined, lifecourse studies would be of tremendous interest which may help formulate precise public health policy: helping to guide appropriate screening, targeted interventions and potential supplementation guidelines.

### Conclusions

This study is the first to combine cross-sectional and longitudinal designs to observationally investigate the utility of the composite score cB_12_, its constituent static/functional markers as well as *HIBCH rs291466* as potential diagnostic and prognostic tools in assessing and predicting vitamin B_12_ status during pregnancy. We highlighted significant changes in vitamin B_12_ biomarker values were found over the course of pregnancy, although the mean/median of these values largely fell within generally recognized normal values in a non-pregnant population. Whether or not these represent optimal levels for risk reduction in pregnancy is yet to be defined. The 5th and 95th percentile cut-offs were used to define a set of “suggested” reference ranges for each of the three trimesters, as ‘normal' is likely to be a dynamic target and despite identifying a subset of women at elevated risk of vitamin B_12_ insufficiency from the first trimester onwards, our analysis did not find that any of the biomarkers individually or grouped could reliably and accurately predict true insufficiency/deficiency in the late stages of pregnancy, at least in a B_12_-replete cohort. Yet, our data provide some indication that HoloTC might be a robust biomarker in this regard which will need to be validated in future studies.

## Data Availability Statement

The original contributions presented in the study are included in the article/Supplementary Material, further inquiries can be directed to the corresponding author/s.

## Ethics Statement

The studies involving human participants were reviewed and approved by Milton Keynes Research Ethics Committee. The patients/participants provided their written informed consent to participate in this study.

## Author Contributions

KA suggested, designed and oversaw the study, and supervision of M-JD. M-JD contributed to planning and implementing all statistical and data analyses, writing, and interpreting the results. TA contributed to the analyses, discussion, and writing. RY contributed to the analysis and writing of the manuscript. MG-M, AS-M, and DH oversaw and completed all the biochemical analyses. All authors helped to interpret results and write the report, and also have seen and approved this manuscript.

## Conflict of Interest

The authors declare that the research was conducted in the absence of any commercial or financial relationships that could be construed as a potential conflict of interest.

## Publisher's Note

All claims expressed in this article are solely those of the authors and do not necessarily represent those of their affiliated organizations, or those of the publisher, the editors and the reviewers. Any product that may be evaluated in this article, or claim that may be made by its manufacturer, is not guaranteed or endorsed by the publisher.
