# Supplementary material for: Evaluating the Diagnostic Value of a Combined Indicator of Vitamin B12 Status (cB12) Throughout Pregnancy
Source: Front Nutr. 2022 Jan 26;8:789357. doi: 10.3389/fnut.2021.789357 (PMC8825834; doi:10.3389/fnut.2021.789357)
Supplement: Supplementary file 1 [file Data_Sheet_1.docx]

**SUPPLEMENTARY MATERIAL**

**Supplementary Table 1 | Vitamin B_12_ biomarker cut-off points for insufficiency used in the current study.**

| *Biomarker* | *Cut- off* | *Rationale for cut-off* | *Evidence* |
| --- | --- | --- | --- |
| HoloTC (pmol/L) | <32 | Early marker for insufficiency; associated with elevated MMA. | Nexo & Hoffman-Lucke (2011) |
| Serum folate  (nmol/L) | <6.8 | Indicative of possible folate deficiency; can be used to rule out lack of folate as a cause of high tHcy levels. | WHO Working Group on Nutritional Anaemias (1968) |
| Serum B_12_  (pmol/L) | <148 | Associated with elevated MMA; widely used as clinical cut-off for deficiency. | Bailey et al (2013) |
| tHcy (μmol/L) | >15 | Indicative of hyperhomocysteinemia. | Myers et al (2009) |
| MMA (nmol/L) | >350 | Associated with low/borderline B_12_ concentrations. | Miller et al (2006) |
| cB_12_ | <-0.5 | “Low” suggested cut-off for researchers and clinicians. | Fedosov et al (2015) |
| *cB_12_, combined B_12_ indicator; HoloTC, holotranscobalamin; MMA, methylmalonic acid; tHcy, total homocysteine.* | | | |

**Supplementary Table 2 | Participant exclusion criteria for the current study further to randomisation in the original SPRINT study.**

| *Criteria* | *Rationale* | *Evidence* |
| --- | --- | --- |
| Samples unavailable for analysis | No data for all three trimesters. | - |
| Diagnosis of diabetes | Potentially prescribed metformin which can lower vitamin B_12_ status. | Liu et al (2014) |
| BMI in the ‘severely obese’ category (>35 kg/m^2^) | Evidence of association between obesity and lower vitamin B_12_ status in pregnancy. | O’Malley et al (2018) |
| BMI in the ‘obese’ category (30-34.9 kg/m^2^) |  |  |
| BMI in the ‘underweight’ category (<18.5 kg/m^2^) | Aims are to study healthy participants so only healthy weight range considered | - |

**Supplementary Table 3 | Characteristics of the candidate SNP investigated in HIBCH (rs291466) and functions of the protein it encodes**

| CHR | Gene | Function | SNP | Position ^1^ | SNP location | Allele, major/minor | MAF, fraction | Genotype count | P-HWE |
| --- | --- | --- | --- | --- | --- | --- | --- | --- | --- |
| 2q32 | *HIBCH* | Hydrolysis of 3-hydroxy- isobutyrate coenzyme-A to its ketone form (2-methyl-3-oxopropano- ate) in the valine degradation pathway | rs291466 | 190319749 | Initiator Codon | G/A | 0.40 | 39/40/16 | 0.59 |

^1^ From the dbSNP database <https://www.ncbi.nlm.nih.gov/snp/rs291466>

*HIBCH*, 3-hydroxyisobutyryl-CoA hydrolase; HWE, Hardy-Weinberg equilibrium; MAF, minor allele frequency

**Supplementary Table 4 | Number of women taking supplements in the placebo arm of the SPRINT study.**

| **Supplements** | **T1** | **T2** | **T3** |
| --- | --- | --- | --- |
| Folic Acid 400 mcg | 81 | 2 | - |
| Folic Acid 800 mcg | - | 1 | - |
| Multivitamin | 30 | 11 | 1 |
| Calcium + Vitamin D | - | 2 | - |
| Vitamin C | 1 | 2 | - |
| Iron + Vit B | 1 | - | - |
| Zinc | - | 2 | - |
| Omega 3 | - | 2 | 3 |
| Vitamin D | - | 3 | - |
| Iron | - | 3 | 1 |
| Vitamin C+D | - | 1 | - |
|  |  |  |  |
| **N** | **113** | **29** | **5** |

**Supplementary Table 5 | Effects of folic acid and B_12_-containing multivitamin supplementation in the first trimester of pregnancy on mean levels of vitamin B_12_ biomarkers in all trimesters.**

|  | **Biomarker** | **Supplementation in T1** | | **P-value** |
| --- | --- | --- | --- | --- |
|  |  | **Folic Acid** | **Multivitamin** |  |
| T1 | Folate (nmol/L) | 13.58 | 15.05 | 0.28 |
|  | sB_12_ (pmol/L) | 328.5 | 315.5 | 0.39 |
|  | HoloTC (pmol/L) | **59.59** | **78.74** | **0.02** |
|  | tHcy (μmol/L) | 5.07 | 4.9 | 0.85 |
|  | MMA (nmol/L) | 161.3 | 143.2 | 0.25 |
|  | cB_12_ | 0.31 | 0.46 | 0.12 |
| T2 | Folate (nmol/L) | **10.2** | **13.15** | **0.001** |
|  | sB_12_ (pmol/L) | 297.4 | 299.9 | 0.65 |
|  | HoloTC (pmol/L) | **63.52** | **68.79** | **0.02** |
|  | tHcy (μmol/L) | 4.5 | 4.1 | 0.32 |
|  | MMA (nmol/L) | 210.5 | 199.8 | 0.06 |
|  | cB_12_ | 0.38 | 0.53 | 0.33 |
| T3 | Folate (nmol/L) | **8.9** | **12.81** | **0.01** |
|  | sB_12_ (pmol/L) | 257 | 259 | 0.60 |
|  | HoloTC (pmol/L) | **57.38** | **66.4** | **0.02** |
|  | tHcy (μmol/L) | 6.26 | 5.056 | **0.03** |
|  | MMA (nmol/L) | 210.5 | 199.8 | 0.62 |
|  | cB_12_ | 0.01 | 0.02 | 1.00 |

Values presented are mean concentrations.

* Significance level P<0.05.

*HoloTC, holotranscobalamin; MMA, methylmalonic acid; tHcy, total homocysteine; sB_12_, serum B_12_*

**Supplementary Table 5. Effects of BMI and age on four biomarkers of vitamin B_12_ status (serum vitamin B_12_, HoloTC, MMA, tHcy), folate, cB_12_ and HoloTC: sB_12_ ^1^.**

Our results showed no effects of maternal age and BMI on any of the biomarkers of B_12_ status and folate in this study cohort which is consistent with previous reports from the literature. It has previously been shown that obese pregnant women (BMI> 30 kg/m2) were at higher risk of B_12_ deficiency. However, findings on the association between sB_12_ concentration and obesity have not been consistent.

|  |  | **Trimester 1** | |
| --- | --- | --- | --- |
|  |  | Age | BMI |
| **Trimester 1** | sB_12_ | 0.12 (0.2) | 0.001 (0.9) |
|  | HoloTC | 0.16 (0.1) | -0.03 (0.7) |
|  | MMA | -0.08 (0.4) | -0.2 (0.05) |
|  | tHcy | -0.17 (0.2) | 0.001 (0.9) |
|  | cB_12_ | 0.14 (0.2) | 0.08 (0.4) |
|  | HoloTC: sB_12_ | 0.04 (0.6) | -0.03 (0.7) |
|  |  | **Trimester 2** | |
|  |  | Age | BMI |
| **Trimester 2** | sB_12_ | 0.15 (0.1) | -0.05 (0.6) |
|  | HoloTC | 0.12 (0.2) | 0.03 (0.7) |
|  | MMA | -0.09 (0.3) | -0.03 (0.7) |
|  | tHcy | -0.09 (0.4) | -0.03 (0.7) |
|  | cB_12_ | 0.17 (0.1) | 0.02 (0.8) |
|  | HoloTC: sB_12_ | -0.04 (0.6) | 0.13 (0.2) |
|  |  | **Trimester 3** | |
|  |  | Age | BMI |
| **Trimester 3** | sB_12_ | 0.11 (0.2) | -0.19 (0.06) |
|  | HoloTC | 0.13 (0.2) | -0.18 (0.07) |
|  | MMA | -0.10 (0.3) | -0.07 (0.5) |
|  | tHcy | 0.10 (0.3) | 0.04 (0.6) |
|  | cB_12_ | 0.12 (0.2) | -0.13 (0.2) |
|  | HoloTC: sB_12_ | 0.02 (0.8) | -0.008 (0.9) |

^1^ Data represents r coefficients from Pearson correlation tests. p-values are represented in brackets. *BMI, body mass index; HoloTC, holotranscobalamin; MMA, methylmalonic acid; tHcy, total homocysteine; sB_12_, serum B_12_*

**Supplementary Table 7 | Longitudinal changes in cB_12_ and MMA by *HIBCH* *rs291466* genotype ^1^**

|  |  | Geometric Mean | | |  |  |  |
| --- | --- | --- | --- | --- | --- | --- | --- |
| Variable change (%) | **Subjects**  ***n*** | G/C | GA/CT | AA/TT | **β ± SE** | ***R^2^*** | **P-value** |
| Change in MMA | 90 | 58.28% | 32.53% | 24.16% | 17.481± 7.443 | 0.05 | 0.02 |
| Change in cB_12_ | 91 | 51.86% | 72.96% | 89.01% | 29.20 ± 47.69 | 0.004 | 0.54 |

^1^ All genetic analyses were carried out with the use of R software using linear regression ***lm()*** function to test the effect of the exposure variable (*rs291466 genotype)* on the outcome variables (% change in MMA and cB_12_ from T1 to T3).

Abbreviations: cB_12,_ combined indicator of B_12_ status; HoloTC, holotranscobalamin; sB_12,_ serum B_12_; tHcy, total homocysteine; MMA, methylmalonic acid.

**Supplementary Table 8 | Suggested reference ranges for biomarkers of Vitamin B_12_ status across the three trimesters of pregnancy, based on data collected from 114 healthy pregnant women who subsequently delivered healthy infants.** Reference range are given for biomarkers of vitamin B_12_ in pregnancy, defined using the 95^th^ percentile cut-off (for homocysteine [tHCy, micromol/L] and methylmalonic acid [MMA, nmol/L]) and 5^th^ percentile cut-off (for transcobalamin [HoloTC, pmol/L], serum folate [Folate, nmol/L], serum B_12_ [pmol/L] and cB_12_ of data collected from 114 healthy pregnant women across the three trimesters (T1, T2, T3) of pregnancy. Values given in *italics* are outside of the ranges suggested by the British Haematological Society for non-pregnant individuals. This may represent either a normal physiological adaptation to pregnancy, or potentially suggest suboptimal vitamin B_12_ levels measured in this cohort.

| Trimester | Folate  nM/L | sB_12_  pM/L | HoloTC  pM/L | tHcy  μM/L | MMA  nM/L | cB_12_ |
| --- | --- | --- | --- | --- | --- | --- |
| 1 | >23.3 | ***>141*** | >35.5 | <6.8 | <258 | >-0.22 |
| 2 | >14.6 | ***>134*** | >35.1 | <6.8 | <342 | >-0.29 |
| 3 | >9.61 | ***>98.2*** | ***>30*** | <9.08 | ***<367*** | >-0.51 |

**Supplementary Table 9 | Concentration levels of markers of B_12_ status, folate (nM/L), sB12 (pmol/L), HoloC (pmol/L), tHcy (μM/L), MMA (nM/L) and cB_12_ for top 5 “more sufficient” mothers.**

*
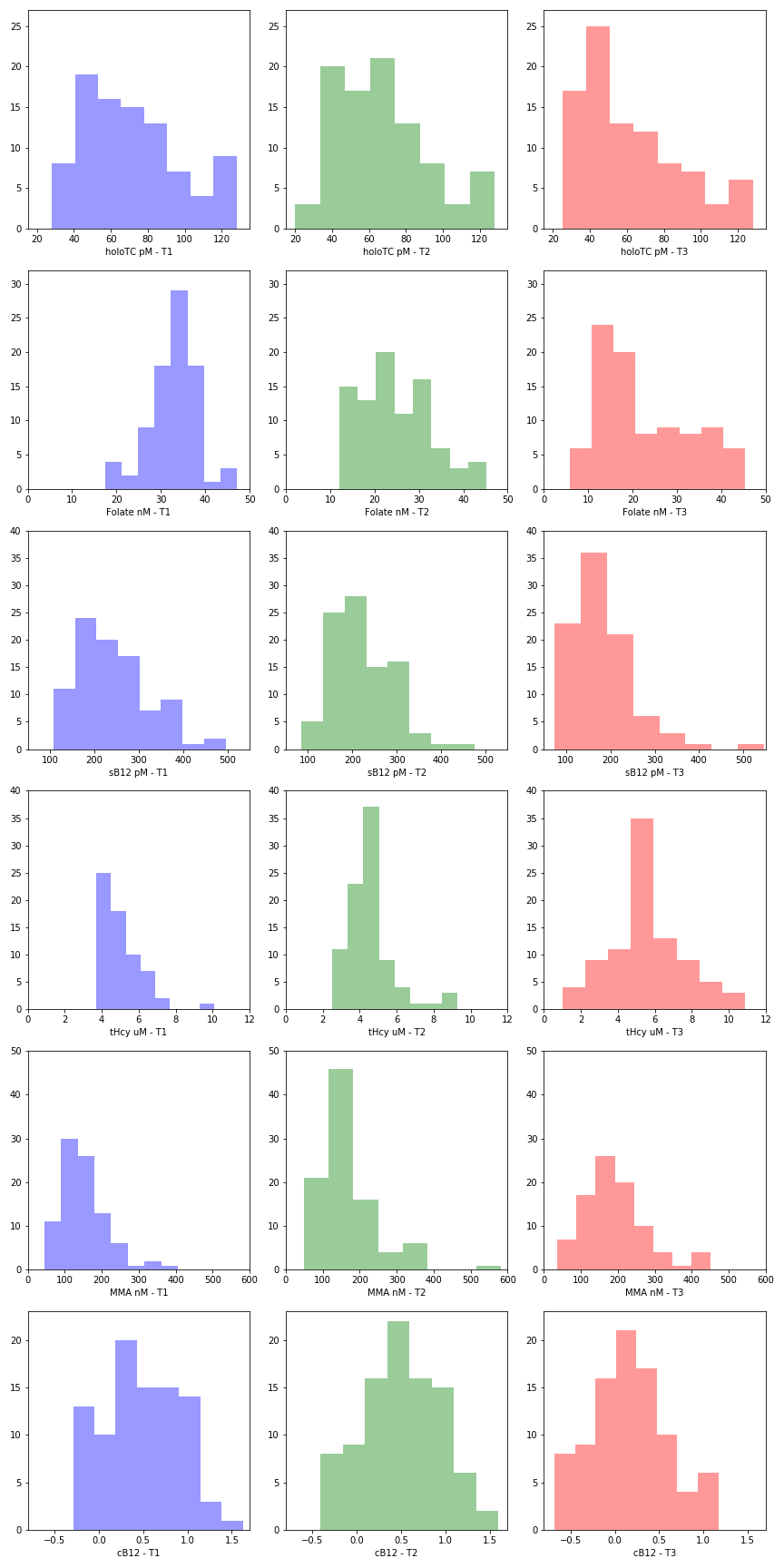
*

**Supplementary Figure 1 | Histograms showing biomarker distributions across each of the three trimesters**

Histograms showing the distribution of data points for each biomarker, transcobalamin [Holo TC, pmol/L], serum folate [Folate, nmol/L], serum B12 [pmol/L], homocysteine [tHCy, micromol/L], and methylmalonic acid [MMA, nmol/L] as well as the score for combined B12 [cB12] across the three trimesters (T1, T2, T3) of pregnancy. Biomarker value is given on the x-axis, count on the y-axis.


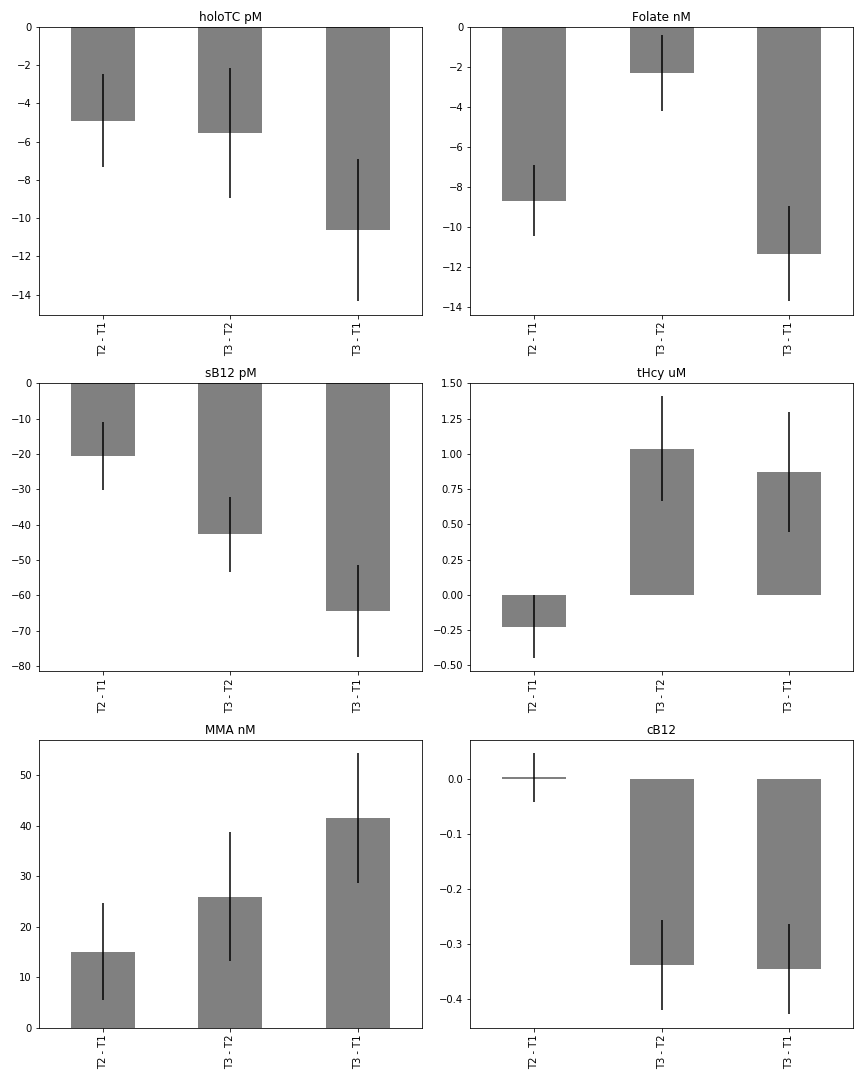


**Supplementary Figure 2 | Average differences, as a percentage of baseline levels, between vitamin B_12_ biomarkers over time.**

Bar chart demonstrating the mean of the differences between individual biomarkers, taken across the three trimesters in normal pregnancy. 95% confidence intervals for these differences are represented by the vertical line. Biomarkers included are transcobalamin [Holo TC, pmol/L], serum folate [Folate, nmol/L], serum B12 [pmol/L], homocysteine [tHcy, micromol/L], methylmalonic acid [MMA, nmol/] and score for combined B12 [cB12]. Results not given for combined B12 score between trimesters one and two, as change in value was not statistically significant.


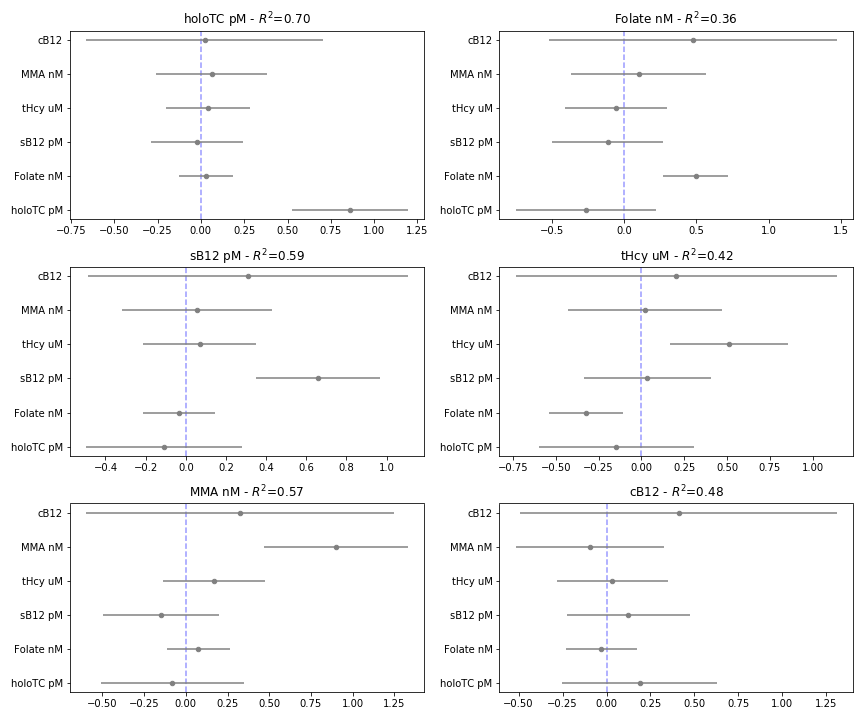


**Supplementary Figure 3 | Regression analysis from the third to the second trimester**

Visualisation of linear regression analysis, showing coefficient and standard errors, examining whether each biomarker [transcobalamin [Holo TC, pmol/L], serum folate [Folate, nmol/L], serum B12 [pmol/L], homocysteine [tHCy, micromol/L], methylmalonic acid [MMA, nmol/L ], plus score of combined B12 [cB12] in the third trimester of normal pregnancy [T3] can be significantly predicted by itself or the other biomarkers measured in second trimester of pregnancy [T2] (given on the y-axis). This was calculated using a standardized linear regression, with 95% confidence intervals on the coefficients.


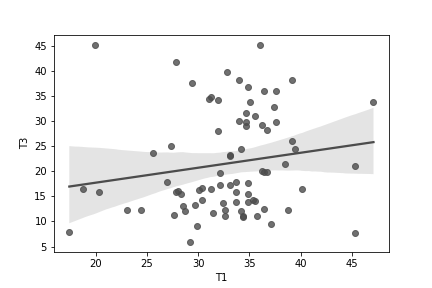

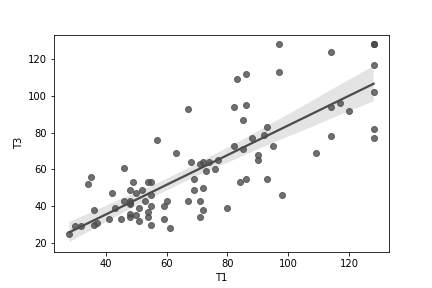


holoTC, T1 – T3, R-squared 0.7

Folate, T1 – T3, R-squared 0.17

**Supplementary Figure 4 | Representation of the strength of the pairwise relationships in regression analysis**

Visualisation of the regression analysis showing the strength of the pairwise relationship when (i) there is a strong correlation (holoTC, R-squared = 0.7) and (ii) when there is a weaker correlation (folate, R-squared = 0.17).


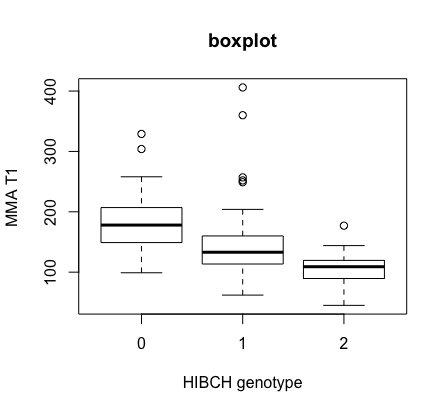

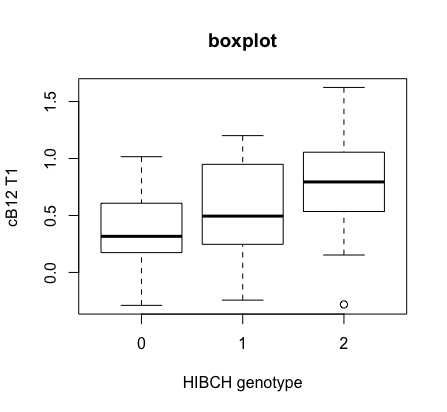


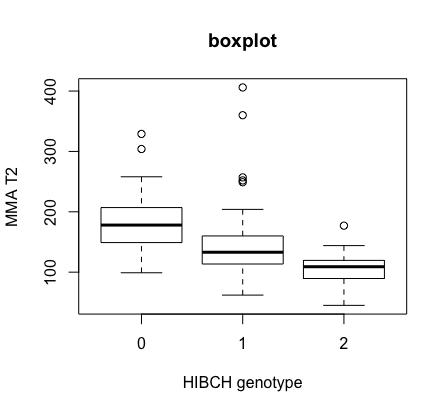

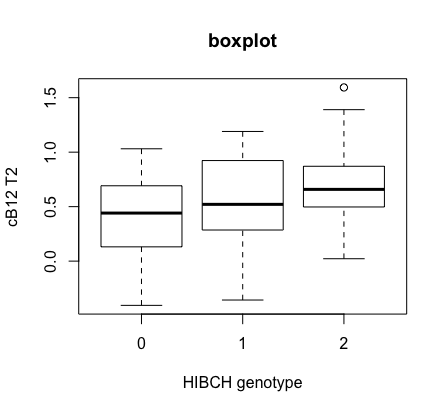


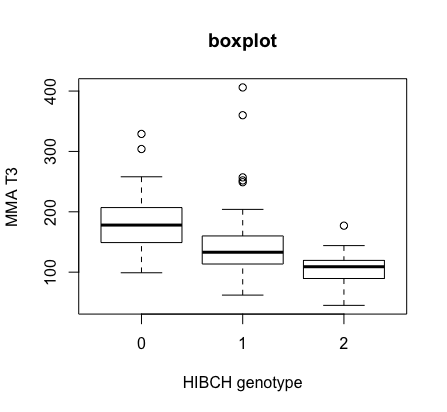

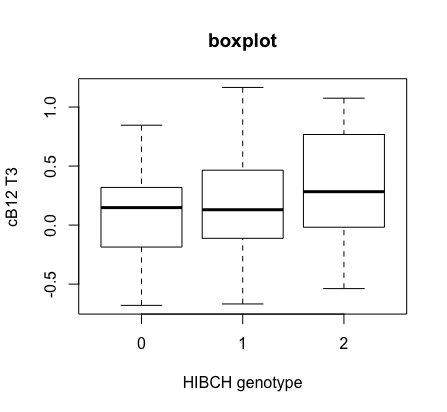


**Supplementary Figure 4 | Boxplots depicting concentrations of MMA and cB_12_ stratified according to HIBCH genotype across all trimesters of pregnancy.**

HIBCH genotypes GG (0) GA (1) and AA (2) are represented.
